# Dietitians use and recommend dietary supplements: report of a survey

**DOI:** 10.1186/1475-2891-11-14

**Published:** 2012-03-14

**Authors:** Annette Dickinson, Leslie Bonci, Nicolas Boyon, Julio C Franco

**Affiliations:** 1 Dickinson Consulting, LLC, 3432 Denmark Avenue, #350, St. Paul, MN 55123, USA; 2 Sports Nutrition, UPMC Center for Sports Medicine, University of Pittsburgh Medical Center, 3200 S. Water Street, Pittsburgh, PA 15203, USA; 3 Ipsos Public Affairs, 1700 Broadway, New York, NY 10019, USA

**Keywords:** Dietary supplements, Supplement surveys, Dietitians' health habits

## Abstract

**Background:**

Dietary supplement use is common in the United States, with more than half of the population using such products. Nutrition authorities consistently advocate a "food first" approach to achieving nutritional adequacy but some, including the Academy of Nutrition and Dietetics (formerly the American Dietetic Association), also recognize that dietary supplements have a role to play in improving nutrient intake to support health and wellness. Surveys show that many health professionals use dietary supplements themselves and also recommend dietary supplements to their patients or clients.

**Methods:**

As one component of a series of surveys of healthcare professionals (the "Life...supplemented" HCP Impact Studies), 300 registered dietitians were surveyed in 2009 regarding their personal use of dietary supplements and whether they recommend dietary supplements to their clients. Respondents were registered dietitians whose business involved seeing clients in a private practice or at a clinic.

**Results:**

Seventy-four percent of the dietitians surveyed said they were regular users of dietary supplements, while 22% said they used dietary supplements occasionally or seasonally. The primary reasons for using dietary supplements were for bone health (58%), overall health and wellness (53%), and to fill nutrient gaps (42%). When asked if they "ever recommend dietary supplements to clients," 97% of the respondents said they did. The primary reasons were for bone health (70%), to fill nutrient gaps (67%), and overall health and wellness (49%). Eighty-seven percent of the dietitians agreed with the statement, "There are gaps in clients' diets that could effectively be addressed with dietary supplements." The dietitians surveyed said they followed healthy habits including eating a balanced diet (96%), managing stress (92%), visiting their own healthcare professional regularly (86%), exercising regularly (83%), maintaining a healthy weight (80%), and getting a good night's sleep (72%). Nearly all respondents (95%) expressed an interest in continuing education about dietary supplements on a variety of topics.

**Conclusions:**

Many dietitians, like other health professionals, use dietary supplements regularly as part of their own approach to a healthy diet and lifestyle. They also recommend dietary supplements to their clients or patients, to promote health.

## Background

Dietary supplement use is common among U.S. adults, according to the National Health and Nutrition Examination Surveys (NHANES), with the prevalence of dietary supplement use in the month preceding the survey reported to be 52% in the 1999-2000 NHANES and 54% in the 2003-2006 NHANES [1,2]. These surveys show that usage is higher among older adults than among young adults, higher among women than among men, and increases as the level of education increases. Most users of dietary supplements say their primary motivation is to improve overall health or wellness or to fill perceived nutrient gaps in their dietary intake [3].

National nutrition surveys show that many Americans fall short in consumption of several vitamins and minerals, including calcium, zinc, magnesium, iron, vitamins A and D, vitamins C and E, and vitamin B-6 [4,5]. The Academy of Nutrition and Dietetics (formerly the American Dietetic Association) urges improvement in overall dietary habits as the primary tool for improving nutrient intake, but the organization's position paper on nutrient supplementation also recognizes that dietary supplements may have a role to play in helping people achieve nutritional goals [6]. The Dietary Reference Intakes established by the Institute of Medicine suggest supplemental intakes of some nutrients for some population groups, such as folic acid for women of childbearing age and vitamin B-12 for people over the age of 50, and the 2010 Dietary Guidelines for Americans also incorporate these recommendations [7,8]. The National Osteoporosis Foundation recognizes that some people may need supplemental intakes of calcium and vitamin D to reach levels considered to be necessary to build and maintain optimum bone mass during growth and early adulthood and to reduce bone loss during aging [9]. Researchers at the Harvard School of Public Health have designed a modified Food Guide Pyramid with a sidebar recommending a "daily multivitamin plus extra vitamin D (for most people)" [10]. Long chain omega-3 fatty acids such as EPA and DHA are consumed at very low levels by most Americans, and increases in intake could improve cardiovascular health and also provide other benefits [11]. Fiber intakes in the U.S. are also low, and fiber supplements as well as consuming more foods high in fiber could be beneficial for a large fraction of the population [12].

While the prevalence of consumer usage of dietary supplements is well documented, less attention has been paid to usage among health professionals and to whether health professionals recommend dietary supplements to their patients or clients. As part of a series of surveys on healthcare professionals' practices relating to dietary supplements, we conducted a survey to examine the extent to which dietitians use dietary supplements or recommend dietary supplements to their clients.

## Methods

The Council for Responsible Nutrition (CRN), a trade association representing the dietary supplement industry, contracted with Ipsos Public Affairs to conduct a series of surveys of healthcare professionals regarding their use of dietary supplements and whether they recommended dietary supplements to their patients or clients. The three phases of the "Life...supplemented" Healthcare Professionals Impact Study (HCP Impact Study) included a 2007 survey of physicians and nurses; a 2008 survey of cardiologists, dermatologists and orthopedists; and this 2009 survey of dietitians, which also included surveys of nurse practitioners and pharmacists (data on nurse practitioners and pharmacists not included in this report). "Life...supplemented" is a consumer wellness initiative funded by a number of CRN member companies and by the CRN Foundation.

The survey of dietitians was administered online between October 3 and October 11, 2009, to 300 Registered Dietitians (RDs) who had an office-based practice in a clinic or in private practice and who reported seeing at least one patient per week at their practice site. The survey sample size was selected to provide 95% confidence that the sampling error would not exceed plus-or-minus 5.7%, and recruitment was discontinued once the desired sample size had been reached. The 300 RDs consisted of 56 members of the eRewards U.S. online panel and another 244 from a database obtained from states' Registered Dietitian licensure records.

The eRewards panel is a U.S. online panel including over 1,500 dietitians who "opted into" the panel in order to take part in market research surveys in exchange for a points-based incentive. A total of 498 registered dietitians who are part of the eRewards panel were invited to take part in the survey, and 207 responded. The response rate of 42% was well within the norms for online surveys conducted among eRewards panel members from this profession. Among those who responded, 27% met all qualifications and completed the entire survey. The remainder consisted of dietitians who did not meet all qualifications necessary to take part in the survey (57%); met the desired qualifications, but failed to complete the entire questionnaire (4%); or did not take part in the survey because the target number of completed interviews had been achieved (12%). Qualified respondents included only those who described their primary occupation as Registered Dietitian and reported seeing clients weekly as part of their practice in an office or a clinic.

To complement the sample, an additional comprehensive database of dietitians originally sourced from states' Dietitians licensure records updated quarterly was also used. A total of 1,700 randomly selected, pre-qualified dietitians from that database were sent an invitation to take part in the survey. As compensation for their participation, they were offered an honorarium of $15. A total of 671 dietitians responded. The response rate of 39% was typical of online surveys among dietitians sourced from this database. Among those who responded, 38% met all qualifications and completed the entire survey, while 49% did not meet all the qualifications needed to take part in the survey, 9% were excluded because the target number of completed interviews had been achieved, and 3% met the desired qualifications, but failed to complete the entire questionnaire. Qualified respondents included only those who described their primary occupation as Registered Dietitian and reported seeing clients weekly as part of their practice in an office or a clinic.

The letters inviting Registered Dietitians from both sample sources to take part in the survey did not identify the subject of the survey. To protect against conflicts of interest, dietitians were not considered eligible for the survey if they were affiliated with a pharmaceutical or dietary supplement company or a market research firm or advertising agency.

The dietitians were asked whether they were "regular" users of dietary supplements. The term "regular" was not defined, except in contrast to other options, which included "occasional" use (taking products throughout the year when they think of it or when the need arises) or "seasonal" use (taking them only during part of the year such as during the cold/flu season or allergy season). Dietitians who described themselves as regular users were asked whether they regularly used a variety of dietary supplements (vitamins, minerals, herbal products, sports nutrition or specialty supplements) or whether they regularly used typically only a multivitamin or multivitamin-mineral. The terms "multivitamin" and "multivitamin-mineral" were not defined in the survey instrument, as these are the accepted terms of commerce used to identify products containing all or most of the 13 vitamins, with or without one or more essential minerals. The dietitians were asked to identify the specific products they used (but not the level or dosage of each component) and to indicate their reasons for using dietary supplements. The respondents were also asked about the number of years they had been taking supplements and whether other members of their household took them.

Dietitians were also asked whether they "ever recommend dietary supplements" to clients, and if so, to specify their reasons for doing so. The reasons for personally using dietary supplements and for recommending supplements to clients were selected by respondents from a list of over 30 options provided in the questionnaire and described in more detail in the Results section of this report. For the most part, the list of options provided in the questionnaire for using supplements and for recommending supplements were the same, but a few (such as questions about men's health issues) appeared only on one list or the other. In addition to the provided options, participants could mention "other" reasons for using and recommending dietary supplements.

Respondents were asked what resources they trusted for reliable information for making decisions or recommendations about dietary supplements. Their attitudes about supplements and about health and wellness were also probed.

## Results

The 300 dietitians who responded to this survey were almost all female (96%), and 84% were in the age range 30 to 59 (27% in their 30 s, 24% in their 40 s, and 33% in their 50 s). They were geographically dispersed, with 37% in the midwestern U.S., 31% in the south, 18% in the northeast, and 14% in the west. Forty-four percent of the respondents had been qualified as dietitians for more than 20 years, 28% for 11 to 20 years, 24% for 4 to 10 years, and only 4% for less than 4 years. Almost three-quarters (74%) were members of the American Dietetic Association (now the Academy of Nutrition and Dietetics). Most of the dietitians surveyed (66%) said they saw 1 to 50 clients per week in an office or clinic setting, but 34% saw a larger number. Table 1 summarizes demographic characteristics and overall patterns of supplement use reported by the dietitians surveyed.

**Table 1:** Demographic characteristics and reported use of dietary supplements by all dietitians surveyed (n = 300)

Characteristic	Description	Percent
Gender	Female	96%

	Male	4%

Age	Under 30	7%

	30 to 39	27%

	40 to 49	24%

	50 to 59	33%

	60 to 69	8%

Region	Midwest	37%

	South	31%

	Northeast	18%

	West	14%

Years qualified in profession	3 years or less	4%

	4 to 10 years	24%

	11 to 20 years	28%

	21 years or more	44%

Clients seen per week	1 to 50	66%

	51 to 100	21%

	Over 100	13%

Use of dietary supplements	Any current use	96%

	Regular use	74%

	Occasional use	20%

	Seasonal use	2%

	Used in the past	3%

	Never used	1%

Duration of supplement use	No current use	4%

	3 years or less	16%

	4 to 10 years	41%

	11 to 20 years	19%

	Over 20 years	19%

Almost all of the dietitians surveyed (96%) said they had used a dietary supplement (regularly, occasionally, or seasonally) during the previous year, as shown in Table 1. There was no significant difference in the overall prevalence of supplement use according to age, region, or years qualified in practice (data not shown). Seventy-four percent of the dietitians in this survey indicated that they were regular users of dietary supplements. This included 36% who said they regularly used a variety of supplements and 38% who said their regular use typically consisted only of a multivitamin or a multivitamin/mineral supplement. Twenty percent said they used supplements occasionally, and 2% said they used supplements seasonally. Three percent said they had used supplements in the past but no longer did so, and only 1% said they had never used dietary supplements. Seventy-two percent of the dietitians surveyed said that at least one other member of their household also used dietary supplements.

Among the dietitians who said they used dietary supplements at least some of the time, 43% had taken them for 4 to 10 years, 20% had taken them for 11 to 20 years, and 20% had taken them for over 20 years. Only 16% had taken them for 3 years or less. The top three reasons for taking dietary supplements were for bone health (58%), for overall health and wellness benefits (53%), and to fill nutrient gaps in the diet (42%).

The prevalence of multivitamin use was high, with 84% of the dietitians saying they had taken a multivitamin within the past year, compared to 63% who had taken calcium, 47% who had taken omega-3 or fish oil supplements, 43% who had taken vitamin D, 29% who had taken vitamin C, 24% who had taken probiotics, 23% who had taken B vitamins, 22% who had taken fiber supplements, 18% who had taken green tea supplements, 13% who had taken flax seed oil, and 11% who had taken glucosamine and/or chondroitin. Table 2 shows the dietary supplements taken during the previous year by more than 10% of the dietitians surveyed.

**Table 2:** Dietary supplements used in the previous year (regularly, occasionally, or seasonally) by more than 10% of the dietitians surveyed

Dietary supplement	Percent using in previous year
Multivitamin	84%

Calcium	63%

Omega 3/Fish oil	47%

Vitamin D	43%

Vitamin C	29%

Probiotics (e.g., acidophilus)	24%

Vitamin B or B complex	23%

Fiber	22%

Green tea	18%

Flax seed oil	13%

Glucosamine/chondroitin	11%

When asked if they "ever recommend dietary supplements to clients," 97% of the dietitians surveyed said they did. There was no significant difference in the prevalence of recommending dietary supplements according to age, region, or years in practice (data not shown). The top seven reasons for recommending dietary supplements, cited by more than 40% of the respondents who recommend supplements to their clients, were bone health (70%), filling nutrition gaps (67%), overall health and wellness benefits (49%), lowering cholesterol (46%), heart health (46%), dietary pattern/vegetarian/vegan (43%), and digestive or gastrointestinal health (39%). Diabetes or glucose control was cited by 27% of dietitians and eating disorders were cited by 19% of dietitians as reasons for recommending supplements; these conditions were not included in the list of potential reasons for dietitians' personal use of dietary supplements. Table 3 shows the reasons common to both lists that were selected by at least 10% of survey respondents for personal use of dietary supplements or for recommending dietary supplements to clients. Reasons cited by 6 to 9% of the respondents included sleep problems, cognitive function, and anti-aging. Reasons cited by 5% or fewer included men's health issues (prostate health or sexual function), depression, stress relief, allergies, musculoskeletal pain, women's health (painful periods, PMS), insomnia, anxiety, headaches, and participation in a clinical trial.

**Table 3:** Reasons for recommending and using dietary supplements, selected by 10% or more of dietitians surveyed, from a provided list of reasons

List of reasons (Provided to survey respondents)	Reason for recommending (n = 300)	Reason for using(n = 287)
Bone health	70%	58%*

Fill nutrition gaps	67%	42%*

Overall health/wellness benefits	49%	53%

Lower cholesterol	46%	16%*

Heart health	46%	25%*

Dietary pattern (vegetarian/vegan)	43%	5%*

Digestive/gastrointestinal health	39%	26%*

Maintain healthy cholesterol	33%	17%*

Women's health (prenatal, pregnancy)	29%	12%*

Immune health	25%	25%

Joint health	22%	15%*

Weight management	17%	6%*

Women's health (menopause)	15%	10%

Skin, hair and nails	15%	13%

Flu/colds	15%	21%

Women's health (other)	14%	14%

Sports nutrition and performance	14%	5%*

Eye health	13%	9%

Energy	12%	15%

Energy balance	10%	6%

Other	10%	5%*

* Indicates a statistically significant difference (p < .05, *x*^2^) in the percent that selected this option as a reason for using *vs *a reason for recommending dietary supplements

The most trusted source of information for dietitians consisted of professional journals, cited by 80% of respondents. Three other trusted sources, each cited by more than 50% of the dietitians surveyed, included clinical studies in scientific journals (72%), clinical guidelines from professional organizations (72%), and continuing education conferences (71%).

Eighty-seven percent of the respondents said their clients are "generally comfortable telling me about their dietary supplement usage." The same proportion (87%) said they agreed with the statement, "There are gaps in clients' diets that could effectively be addressed with dietary supplements." However, only 23% of the dietitians in this survey believe that their clients have a good understanding of the appropriate daily intake for dietary supplements.

While only 5% of the dietitians surveyed said they personally used dietary supplements for reasons related to sports performance, 14% of them said they sometimes recommended supplements for sports-related reasons, and 46% expressed an interest in continuing education on this topic.

The dietitians surveyed had a very high level of adoption of healthy habits, including trying to eat a balanced diet (96%), managing stress (92%), visiting their own healthcare professional regularly (86%), exercising regularly (83%), maintaining a healthy weight (80%) and regularly getting a good night's sleep (72%). Only 24% said they often consumed large quantities of caffeine, and very few smoked (3%) or consumed large quantities of alcohol (3%).

Nearly all respondents (95%) expressed an interest in continuing education (CE) about dietary supplements on a variety of topics. The specific subjects mentioned by more than 40% of the dietitians interested in CE about dietary supplements included: interactions of drugs and dietary supplements (74%), basics about dietary supplements (72%), how to counsel clients about dietary supplements (70%), weight management (68%), women's health (65%), heart health (65%), drug induced nutrient depletion (56%), dietary supplement regulation (54%), and sports nutrition supplements (48%).

## Discussion

Dietitians are uniquely qualified to evaluate the adequacy of nutrient intake and to make rational choices about dietary supplement use for themselves and for their clients or patients, when appropriate. While authoritative groups consistently encourage a "food first" approach to achieving nutrient adequacy, it is also recognized that most people have dietary intakes that fall short in some respects and that dietary supplements can make a contribution toward achieving nutritional goals [4-12].

The prevalence of regular dietary supplement use among dietitians (74%) is comparable to that reported in some surveys of the general public and of health professionals. In NHANES 2003-2006, the overall prevalence of dietary supplement use in adults was 54%, but it was 55% in women 31-50 and 72% in women 51-70 [2]. Since the query in NHANES relates to supplement use within the previous month, it captures mostly regular users, so the appropriate comparison within our survey is with regular users. In a large multiethnic cohort in the general population, 58% of men and 72% of women said they used one of eight dietary supplements regularly, defined as at least once a week [13]. Two surveys of health professionals enrolled in an online course on dietary supplements reported levels of usage in excess of 80% [14,15]. In the "Life...supplemented" HCP Health Impact Studies conducted in 2007, 2008, and 2009, regular dietary supplement use was reported for over 70% of dietitians and nurse practitioners; around 60% of pharmacists, nurses and dermatologists; about 50% of family care physicians, ob/gyn physicians, and orthopedists; and 37% of cardiologists [16,17].

It is notable that 96% of the dietitians we surveyed reported using dietary supplements at least occasionally or seasonally if not regularly, and 97% said they "ever recommended" dietary supplements to clients. Our survey is not representative of all dietitians, as it focuses only on a particular segment of dietitians, namely those who see clients in their office or clinic on a regular basis. Presumably the clients come with questions or issues to be addressed, and dietitians engaged in counseling such clients may be more likely than dietitians in other practice settings to recommend specific actions, including the use of dietary supplements.

The top three reasons for using and recommending dietary supplements were the same in this sample of dietitians: bone health, filling nutrition gaps, and overall health/wellness benefits. All three of these issues are relevant to dietitians as well as to their clients. Bone health was cited by 58% as a reason for personal use and by 70% as a reason for recommending dietary supplements to clients. Calcium and vitamin D supplementation are recognized as appropriate for bone health, for people who do not consume adequate amounts of these nutrients from food, and counseling by dietitians has been shown to improve compliance with calcium recommendations by women with low bone density [9,18].

Overall health and wellness was reported to be the reason for using dietary supplements by 53% and the reason for recommending dietary supplements by about the same proportion (49%). Filling nutrient gaps in the diet was the reason given for personal use by 42%, but was cited by 67% as the reason for recommending dietary supplements, likely reflecting the fact that clients may be less knowledgeable than dietitians about planning for adequate dietary intakes. Other reasons cited by more than 40% of respondents for recommending supplements included purposes related to helping protect against disease, including lowering cholesterol (46%) and supporting heart health (46%), although the dietitians were less likely to personally use dietary supplements for these reasons.

Dietary pattern (vegetarian or vegan) was cited by 43% of dietitians in this survey as a reason for recommending dietary supplements but by only 5% as a reason for personal supplement use. The prevalence of this issue as a basis for recommendations to clients is probably related to the fact that there are recognized potentials for nutrient shortfalls in such diets, and vegetarians and vegans are often advised to seek the counsel of a dietitian to ensure against inadequate intakes of nutrients such as vitamin B-12 [19]. Women's health (prenatal, pregnancy) was cited by 29% of dietitians as a reason for recommending dietary supplements, but by only 12% as a reason for personal use of supplements, likely reflecting differences in age between the dietitians and some of their clients. Almost 2/3 of the dietitians surveyed (65%) were age 40 or older. Health care providers generally recommend a prenatal multivitamin supplement during pregnancy, and CDC recommends a multivitamin with folic acid for women of childbearing age to reduce the risk of having a baby with a neural tube birth defect [20].

Some of the dietitians in this survey (14%) indicated that they sometimes recommended dietary supplements to clients for reasons related to sports nutrition, though only 5% selected sports nutrition and performance as a reason for personal supplement use. There are published recommendations available to guide dietitians in advising clients on issues relating to sports nutrition [21,22]. The U.S. and Canadian dietetic associations, jointly with the American College of Sports Medicine, have issued a position statement noting that optimal nutrition can enhance sports performance as well as recovery from exercise and have evaluated the potential role of some supplements as well as dietary habits [22].

Dietitians can help their clients evaluate nutritional needs and select rational dietary supplements. Recognizing that there are quality issues with some products, dietitians could direct clients toward companies with a reputation for quality or toward products bearing a quality seal.

## Conclusions

Dietitians, like other health professionals, use dietary supplements themselves and also recommend dietary supplements to their patients or clients. The top three reasons for personally using dietary supplements and for recommending them to clients were the same and included bone health, overall health and wellness, and filling nutrient gaps in the diet. Some other reasons were more commonly cited as the basis for recommending dietary supplements, rather than reasons for personal use, likely due to the differing conditions and needs of clients. These included reasons relating to special diets, pregnancy, and sports nutrition.

## Abbreviations

CRN: Council for Responsible Nutrition; HCP Impact Study: "Life...supplemented" Healthcare Professionals Impact Study; NHANES: National Health and Nutrition Examination Survey; RDs: Registered Dietitians.

## Competing interests

AD is a consultant to the Council for Responsible Nutrition (CRN) and was formerly a VP and President of the association. LB has consulted with CRN regarding the "Life...supplemented" wellness program. NB is SVP and JF is Senior Research Manager for Ipsos Public Affairs, which conducted the survey for CRN.

## Authors' contributions

AD prepared the original draft of the article, for subsequent evaluation and elaboration by all of the authors working collaboratively. NB and JF participated in the design and administration of the survey, including the data analysis. All of the authors provided meaningful insight regarding the results and implications of the survey findings, in the context of previously reported research, and all approved the final version of the article.
